# Improving Interviews with Children in Abuse Cases: Current Perspectives from Police and Forensic Interviewers

**DOI:** 10.3390/bs16040592

**Published:** 2026-04-15

**Authors:** Pantxika Victoire Morlat, Laurence Alison

**Affiliations:** Department of Psychology, University of Liverpool, Liverpool L69 7ZA, UK

**Keywords:** child investigative interview, victim interview, child abuse, technology-assisted abuse, rapport building, police practice, forensic interviewer

## Abstract

Investigative interviewers play a crucial role in eliciting information from children; therefore, gathering the views and experiences of professionals helps deepen our understanding and guide areas for improvement. Twelve police officers and forensic interviewers, based in the United Kingdom and the United States, were semi-structure interviewed. A thematic analysis was used to analyse the data, leading to the identification of three main themes: *challenges and limitations in interview process*, *strategies for enhancing interview quality* and *effective techniques for information gathering.* Participants noted limited flexibility with respect to minors, technology-related gaps and the significance of third-party disruptions. They called for better training and interview environments with adaptations to fit to children’s variable abilities, sustained rapport and supportive cues. The findings strengthen our understanding of child investigative interviews by providing updated evidence from the professionals who work directly with them. Drawing on the study’s findings, hypotheses were formulated to assess the effectiveness of interview techniques, update guidelines and ultimately improve child protection through more efficient pursuit of justice.

## 1. Introduction

‘…although the chronological age of the child will inevitably help to inform the judicial decision about competency, in the end the decision is a decision about the individual child and his or her competence to give evidence in the particular trial.’ From the Court of Appeal judgment in R v B [2010] EXCA Crim 4.

(extracted from Achieving Best Evidence [ABE] guidelines; Ministry of Justice, 2022)

While the responsibility is often placed on the child to provide sufficient evidence during investigative interviews, issues can also arise from the use of improper interviewing techniques (Lamb et al., 2008). Thus, investigative protocols such as the National Institute of Child Health and Human Development (NICHD) Standard Protocol (SP; Lamb et al., 2008) were developed in the United States (US) to maximise the amount of reliable information from child interviewees and the adoption of appropriate techniques by interviewers. When interviewing children in abuse cases, several challenges often need to be surmounted by interviewers to gather the truth (Henderson & Lamb, 2019; Hershkowitz et al., 2006; Miragoli et al., 2017; VanMeter et al., 2023). As a result, structured forensic interview protocols were developed to guide practitioners toward developmentally appropriate and non-suggestive questioning strategies. The initial version of the NICHD protocol lacked emphasis on rapport-building and emotionally supportive interviewer behaviour, which contributed to the development of the Revised Protocol (RP; Lyon, 2014). The RP provides a ‘friendlier’ version of the pre-substantive phase (development of rapport) and includes guidance for interviewers on using non-suggestive supportive comments, especially in response to reluctant displays (e.g., *And then what happened?*). The 10-Step Investigative Interview is widely used in child investigative interviewing (see Lyon, 2021). In the context of investigative interviewing, rapport is defined as positive interpersonal interactions that enhance the trust of the interviewee towards the interviewer and thus increase cooperation (Abbe & Brandon, 2014). Although the end goal is clear, the techniques used to establish an efficient rapport between children and interviewers can be difficult (Cassidy et al., 2020). In England and Wales, this process is guided by the Achieving Best Evidence (ABE) guidelines, which are used during child investigative interviewing. Like the other protocols, the ABE recommends rapport-building with the child, the use of open-ended questions and the avoidance of leading prompts (Ministry of Justice, 2022). Recently, the United Kingdom (UK) Council for Internet Safety developed guidelines aimed at challenging victim-blaming language and behaviours when dealing with the online experiences of children and young people (UK Government, 2022). This range of guidelines used in the US and the UK provides the professional context in which this study’s participants worked as forensic interviewers and police officers at the time of the data collection.

Rapport-building is widely recognised as a key component of effective investigative interviewing across populations (Alison & Alison, 2017; Alison et al., 2014; Humann et al., 2023; Kim et al., 2020; Surmon-Böhr et al., 2020). Finding ‘effective’ measures suggests adopting techniques that are successful when deployed in the field for what they are intended to do for a specific population (Wilson & Lipsey, 2001). In this context, interview effectiveness can refer to the extent to which investigative techniques facilitate the retrieval of accurate, detailed and legally relevant information from child interviewees. Goal-oriented behaviours, such as getting to know children during the introductory phase of an investigative interview (often defined as ‘narrative practice’) before asking about the alleged abuse, are well-known techniques to establish rapport. This process is believed to promote a friendlier environment (positive elements for rapport-building) whilst preparing children to provide contextual details and priming them for open-ended questions during the substantive phase (Lyon, 2014). Indeed, discussing non-threatening topics can help children feel more at ease before discussing difficult experiences (Brown et al., 2013). Furthermore, effective rapport-building can improve reliability of statements by increasing report accuracy, completeness and resistance to misleading questions (E. A. Price et al., 2016).

Because of age maturation and the impacts of victimisation (e.g., fear, shame) and possible psychotraumatic consequences (e.g., denial, normalisation of abuse), many child victims are reluctant to report abuse, even when the allegations are valid (Hershkowitz & Lamb, 2020). Therefore, the role of the interviewer is to ask questions that would allow children to feel more comfortable disclosing information. Structured interviewing protocols are designed to influence several psychological processes that underpin children’s testimonies. Research has characterised high-quality interviews by the amount and depth of detail provided by interviewees (Johnson et al., 2021). Employing effective processes can enhance both the quantity and relevance of information reported by children, increasing the likelihood that their accounts are coherent and legally useful. As is often the case, only a ‘Yes’ will not be considered sufficient to base an allegation on (H. L. Price & Roberts, 2007). In psychology research, this helps develop understanding of participants’ experiences, perceptions and realities. For instance, not all children understand that the world they experience is not identical to others; this notion is illustrated by the famous Three Mountain Task of Piaget and Inhelder (1967) in which young children (pre-operational stage: two to seven years of age) have difficulty in seeing different viewpoints from their own which can result in interactions with interviewers being challenging. This is further compounded by a lack of material or physical evidence, further emphasising the need for efficient rapport-building for stronger reliability (Klein et al., 2020). Accordingly, in professional guidance such as the ABE guidelines (Ministry of Justice, 2022), the presence of parents/legal guardians in the interview room was deemed beneficial to serve as ‘Interview Supporters’. Although the amount of research on the effect of third parties’ presence during child interviews is relatively small, previous work contradicts official guidelines by recommending that only the child and interviewer should be present (American Professional Society on the Abuse of Children [APSAC] Taskforce, 2012; Gardner & Randall, 2012; Korkman et al., 2024). While these factors highlight longstanding challenges in obtaining reliable testimony from minors, emerging issues should be noted, considering children’s changing social environments.

An emerging area of concern relates to the increasing role of digital environments in children’s lives. Young people’s extensive use of smartphones, social media and online platforms has created new contexts in which victimisation can occur, including online harms, grooming and exploitation (Presta et al., 2024). These forms of abuse may present unique challenges for interviewers, as children may need to describe online interactions, digital communication or experiences occurring across virtual environments. A general increase in digital device use was reported due to the lockdown measures during the COVID-19 pandemic (Wiederhold, 2020). In fact, longer screen time was found to negatively influence cognitive (Short et al., 2018), socio-relational and physical growth during childhood, while increasing neurological and behavioural difficulties (Lissak, 2018; Presta et al., 2024). Children’s digital technology use is also negatively correlated with their well-being and social and behavioural functioning (Harverson et al., 2025), and can reduce conversational engagement (Misirli et al., 2025), an important aspect in the rapport-building process. Indeed, digital technology may influence how children communicate and interact socially, which may also shape their engagement during investigative interviews. While efficient rapport-building is crucial for fostering cooperation in child investigative interviews, professionals must continue to effectively adapt to differences between generations, such as those presented by digital technology.

In summary, the progress of a criminal investigation involving a child generally depends upon (a) the child’s account of the incident and (b) the interviewer’s ability to obtain information from the child. Child victims (under 18 years old) are defined as vulnerable due to their age and thus require adapted measures (Ministry of Justice, 2022). Whilst multiple official guidelines exist, police officers still report difficulties in obtaining accurate accounts during child interviews (Cassidy et al., 2020). Therefore, the aim of this study was to explore practitioners’ perceptions of effective and ineffective techniques, as well as perceived areas for improvement in child investigative interviewing. Through qualitative interviews with police officers and forensic interviewers working with child victims, the study intends to provide insight into how professionals understand the challenges of interviewing children within contemporary investigative contexts. These insights may inform future research and the development of evidence-based interviewing practices.

Overall, gathering practitioners’ thoughts and opinions can help update the existing guidelines and recommend future practice to (i) increase the amount of relevant information gained during criminal investigations, leading to (ii) better protection of children from future victimisation and (iii) improved prosecution of offenders.

## 2. Materials and Methods

### 2.1. Reflexivity Statement

With the topic of interest relating to sensitive topics, it is important to note the expertise of the researcher who conducted and analysed the interviews. At the time of the investigation, P.V.M. had graduated from an MSc in Investigative and Forensic Psychology, obtaining the accreditation from the British Psychological Society (BPS) of Stage 1 in Forensic Psychology as well as a Graduate Membership of the BPS. During the data collection of this study, P.V.M. was pursuing a PhD in Psychology under the supervision of L.A. P.V.M. was awarded Associate Fellow of the Higher Education Academy in relation to her teaching experience to psychology university students, including teaching research methods and statistics. To enhance the credibility and rigour of the analysis, theme development was discussed regularly with the second author. L.A. is a Professor in Psychology and Chair in Investigative and Forensic Psychology. L.A. has over thirty years of experience working on applied projects for law enforcement and the security services as an expert in investigative interviewing techniques. Discussions between the authors involved critical reflection on interpretations and refinement of themes until agreement was reached. An audit trail of coding decisions was maintained throughout the process to support transparency.

The interview schedule was carefully designed to ensure that the questions reflected contemporary interviewing practices. Additionally, a reflexive diary was also used by the lead author to record personal accounts while interviewing participants, and to reflect on the development of understanding of participants’ insights (see Appendix A). This step was believed to be necessary in order to favour trustworthiness and enhance integrity in the researcher’s role (Finlay, 2002).

### 2.2. Participants

Participants were recruited through purposive expert sampling, based upon their expertise in relation to child investigative interviewing, to ensure detailed information about the phenomenon of interest (Etikan et al., 2016). The authors contacted national law enforcement institutions in the UK to advertise the study to their employees via emails, and further obtained help from a child centre located in the US to upload the study’s details onto their organisation website. In total, 12 adult individuals from various law enforcement and child protection agencies participated. The sample included seven forensic interviewers (all females) from the US and five police officers (three males and two females) from the UK with years of experience in child investigative interviewing. While these groups operate within different legal and professional contexts, they were included deliberately to capture a broader perspective on child interviewing practices as the focus of this study was on exploring professionals’ experiences and perceptions in Western anglophone countries, rather than making direct cross-country comparisons. Including both groups provided a richer understanding of shared challenges, enhancing the transferability of the findings for those practising in the field. All participants provided in-depth data, and all contributed to the development of the results. The number of interviewed participants is consistent with qualitative research guidelines recommending between 5 and 25 participants for phenomenological inquiry (Creswell, 2012). Data collection continued until thematic saturation was reached, defined as the point at which no new themes relevant to the research question were identified in the interview transcripts. None of the participants were compensated for their participation.

### 2.3. Qualitative Approach

Mertens (2010) argues that the qualitative method is a type of methodology that allows uncovering details of a specific topic. Since child investigative interviews remain a topic that require ‘*future research […] about the ways in which vulnerable groups are interviewed and how these groups of witnesses provide information when interviewed about their abuse experiences*’ (Johansson et al., 2018, p. 134), adopting a qualitative approach remains favourable for a richer understanding of the unknown. Nonetheless, awareness of the limitations posed by the qualitative design and the lack of data triangulation is acknowledged in the Discussion. The interviews were coded using a latent thematic analysis (TA; Braun & Clarke, 2006). Given the exploratory aim of the study, TA was considered appropriate for identifying recurring perspectives across participants while preserving interpretive depth, in addition to the known efficacy of TA in semi-structured interviews (Clarke & Braun, 2017), and consistency in forensic psychology research (Banister, 2017). Using TA gave an opportunity to the researchers to build a bridge between qualitative and quantitative approaches of the data by developing a qualitative analysis within (post)positivist standards (Willig & Stainton-Rogers, 2017). Indeed, TA is often characterised by uniting quantitative (positivist) and qualitative (interpretative) concepts (Boyatzis, 1998). All participants were asked questions focused upon (A) interviewer’s abilities, (B) environmental aspects of an interview, (C) time considerations during an interview, (D) impacts of adult’s presence on child interview’s quality and (E) child victim’s characteristics.

### 2.4. Design and Procedure

An electronic information sheet including the study aims, data protection information and researchers’ contact details was provided to the participants, and if they agreed to take part, an electronic consent form was sent. Interviews ranged from 24 min to 65 min (*M_Time_* = 42.23 min) and followed an interview structure. As advised by Israel and Hay (2012), despite interviews being semi-structured, an interview structure should be developed in research involving criminal justice to meet appropriate ethical conduct. Remote interviews seemed the most economical and practical option for participants due to the geographically diverse participants. Throughout the process, participants were reassured of their anonymity and were informed that any information regarding institutions/colleagues was voluntarily excluded in the transcript to avoid participants’ concerns regarding the consequences of their statements (e.g., highlighting problems in police practice), and to increase the chances of truthful accounts. An electronic debriefing form with contacts of mental health helplines was provided once the interview ended. The researchers’ contact information was provided to all participants should they experience any distress or emotional strain related to the research topic.

### 2.5. Data Analysis

Each interview was attended by a research participant and the lead author. Interviews were conducted on video or audio-only calls (using Microsoft Teams), recorded, transcribed (verbatim) and anonymised using Microsoft Word. Analysis was supported by NVivo 12 software. An observational coding scheme was followed based on the six-phase guide of Braun and Clarke (2006) (see Table 1). Different themes were developed based upon the reported effective and ineffective techniques and areas for improvement for child investigative interviews. Participants were not consulted during the data analysis.

**Figure 1:**
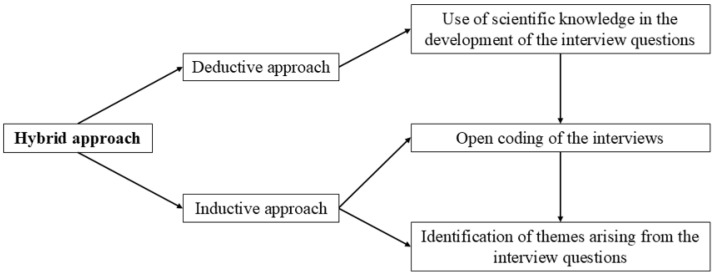
Method of using hybrid approach for qualitative interviews.

Researchers based their coding on a transformative ethical perspective aimed at collaborative work between governmental bodies and researchers (Mertens, 2021), through a hybrid approach of inductive and deductive coding (Creswell, 2012; Fereday & Muir-Cochrane, 2006; see Figure 1). A hybrid approach to qualitative interviews, incorporating both inductive and deductive methods (Braun & Clarke, 2006), helped establishing credibility and trustworthiness (key components of interpretive research; Koch, 1994), while remaining committed to the transformative goal of enhancing social justice by contributing to scientific knowledge on interviewing children during criminal investigations.

Indeed, the transformative perspective emphasises the ethical responsibility of the researcher to challenge inequities, particularly those affecting vulnerable communities (Mertens, 2020), such as child victims. Interview questions were developed based on existing knowledge of child interviews and possible factors that could impact their process. Developing interview questions without any knowledge on current child interview guidelines would not align with the aims of the research, as this study is aimed to pinpoint specific aspects that work, do not work or require improvement.

## 3. Results

**Figure 2:**
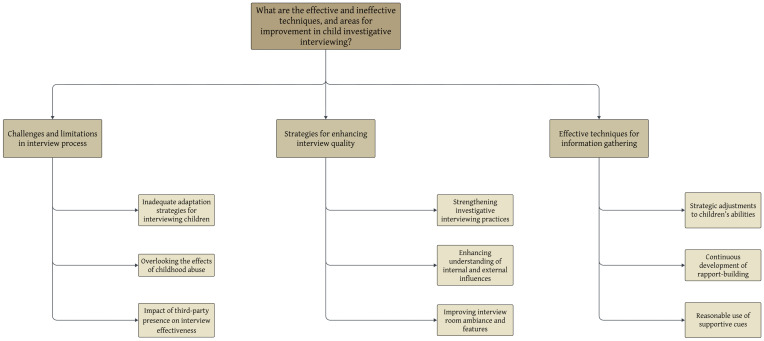
Flowchart of identified main themes and sub-themes.

The current study explored effective and ineffective techniques, as well as areas for improvement in child investigative interviewing. Thematic analysis identified three main themes, each with three sub-themes, based on the entirety of the participants’ responses (see Figure 2).

### 3.1. Theme One: Challenges and Limitations in Interview Process

The use of specific question types was discouraged by participants, and instead they emphasise the importance of providing children with the opportunity for free recall to develop a more robust interview for court proceedings. Another ethical aspect to consider is failing to acknowledge the impacts of abuse on children’s reactions and capacities. For example, children may normalise the abuse or may struggle to discuss it due to the event often being traumatic. Moreover, participants commented on the current policy in England and Wales^1^ in which a parent/legal guardian can assist the interview as ‘Interview Supporters’, which was criticised due to the potential impacts on the child’s willingness to disclose, as well as calling into question the credibility of the information obtained.

#### 3.1.1. Sub-Theme One: Inadequate Adaptation Strategies for Interviewing Children

Due to an often-limited timeframe to interview victims, not falling into the trap of favouring closed-ended questions over open-ended questions seems paramount:

*100% I feel like open-ended questions are more effective than closed-ended questions […] And because I ask very open-ended questions, I’m not putting words in their mouth or telling them what to say. I’m just giving them the opportunity to talk.*

(P4, Female, US, Forensic Interviewer [FI])^2^

In fact, leading questions could be considered unethical and not be well received in court, which may further affect case proceedings and the victims’ justice:

*Then the research shows that [INT: Umm] the [the] more direct questions you ask, the more leading you’re gonna get and the more in trouble like, the more, the less quality interview that prosecution has to rely on so […] I know the research says that typically, any typically over 10 years old is about a suggestible as an adult, but under 10, they’re more suggestive. So, that’s why you want to keep yourself out of it. You want to not suggest things as much as possible and [and] really remove yourself from the evidence that you’re gathering.*

(P7, Female, US, FI)

#### 3.1.2. Sub-Theme Two: Overlooking the Effects of Childhood Abuse

Abuse may be traumatic, and reporting can bring back emotionally dense memories; therefore, participants commonly advised to not dismiss the impacts of abuse on children’s ability to disclose:

Well, I think it’s [it’s] definitely helpful to know about trauma. Like when I trained for this, they didn’t really teach us about trauma. You know, it’s, it wasn’t part of like, the, I mean, they talked about it a little bit, but we’ve [we’ve] studied things by like *NAME* and different like psychiatrists that talk about trauma and the effects [the effects] of it on the brain and how it affects memory, things like that. So, that’s definitely important to know.

(P5, Female, US, FI)

Indeed, it is common for victims to normalise the abuse which can influence the questioning process and obtention of relevant information to protect victims and prosecute alleged offenders:

*I think some of the hardest interviews are the ones where like a teenage girl views that she’s in a romantic relationship with an adult male […] Has anything happened that we need to talk about? Like those questions, they just don’t get it. And it’s also problematic for the chronic grooming kids for who it’s been so normalised. Those normal screening questions of has anything happened that made you feel uncomfortable? Anything that happened that you don’t think was right? Anything happened to your private parts? Those questions, they’re just like, no, no, no, no. And it’s how do I [how do I] get there when they don’t view it as something that was wrong?*

(P8, Female, US, FI)

In addition, victims may fear being judged for their behaviours, showing signs of embarrassment, an aspect that should not be overlooked by interviewers for an increased trustful and reassuring interview environment:

*I think there’s a lot of pressure as well on now thinking [INT* 3 : Ohh] I’ve seen this on [on] *NAME* or a Tele programme and a trial and I shouldn’t say that because they’ll rip us apart and that I shouldn’t say I was drunk because of this. And I think that’s sometimes a barrier and I think it’s really important that we have the conversation with people before that, it doesn’t matter how drunk you were, it doesn’t matter what drugs you’re taking, it doesn’t matter […] Because I think sometimes the worries, they’re just worried that they’re going to be ripped apart in court.

(P11, Female, UK, Police Officer [PO])

#### 3.1.3. Sub-Theme Three: Impact of Third-Party Presence on Interview Effectiveness

The presence of other individuals in the interview room may drastically affect the interview process and hinder responses from children:

*We call it the kiss of death if somebody is in the interview because we can’t control what they’re doing or saying.*

(P8, Female, US, FI)

Especially, parents/legal guardians of the victims may intrude either directly or indirectly in the child’s willingness to disclose abuse:

*Would [would] not want a parent in the room whilst the child was being interviewed […] I think parents may struggle to sit and not be able to intervene or not be able to encourage, not be able to lead a child, I think [INT: yeah].*

(P2, Male, UK, PO)

These accounts suggest that several challenges and limitations exist in child interview processes within abuse investigations. Participants emphasised that effective interviews rely on allowing children opportunities for free recall through open-ended questions rather than closed or leading questions, which may reduce the quality and credibility of evidence in court. This notion is supported by the NICHD Protocol (Lyon, 2014), favouring free recall through the use of open-ended questions (e.g., *What happened?*, *Tell me more*, *What happens next?*), and the avoidance of yes/no or forced-choice questions; the ABE guidelines (Ministry of Justice, 2022) state that ‘Where it is necessary to ask questions, they should, as far as possible in the circumstances, be open-ended or specific-closed rather than forced-choice, leading or multiple’. Interviewers must also recognise the psychological effects of trauma and abuse, as children may normalise abusive experiences, feel embarrassment or fear judgement, all of which can affect disclosure. As advised in the ABE guidelines (Ministry of Justice, 2022): ‘Interviewers must also consider the possible impact on the child of one or more of the following that the child may have experienced: abuse, neglect, domestic abuse and discrimination based on race or disability. There is no single ‘diagnostic’ symptom’. Therefore, a lack of trauma-informed training may limit interviewers’ ability to appropriately support victims. Additionally, the presence of third parties, particularly parents or legal guardians acting as ‘Interview Supporters’, has been criticised and contradicts current official guidelines. Such presence may influence children’s responses or reduce their willingness to disclose sensitive information, potentially compromising the reliability of the interview and the pursuit of justice.

### 3.2. Theme Two: Strategies for Enhancing Interview Quality

Participants suggested some areas for improvement, such as longer and more specialised trainings. Furthermore, they suggested that current guidelines, like the ABE (Ministry of Justice, 2022), should be updated and specifically aimed at improving understanding of rapport-building techniques, increasing awareness of minors’ needs and enhancing understanding into the influence of digital technology on youth. Lastly, the features and ambiance of an interview room were a priority for most participants due to the influence they have on the interview process.

#### 3.2.1. Sub-Theme One: Strengthening Investigative Interviewing Practices

Practising interview techniques with colleagues appears to be an effective strategy for enhancing confidence during the interview process:

…it’s always helpful to spend more time going over the practical aspects and actually being able to feedback having done one [*interview*] then you [you] feedback when you do another and then, you know, perhaps you do a third […] that would probably be why that would have the biggest impact I would say on improving people’s techniques […] having the time to really [really] drill down and get that feedback and act on that feedback and, you know, have a chance to sort of, you know, think about it [INT: Umm] essentially reflect on that feedback as well is really [really] important.

(P12, Male, UK, PO)

Participants particularly commented on the need to further develop interview trainings in terms of length:

I was able to observe when I started here my coworkers doing interviews, and that was helpful for me, you know, and then they would watch mine and give me feedback on how I was doing, things like that. And we’d meet before the interview ended, things like that. So, that was helpful for me. And that, like, the state of *LOCATION*, one had a role-playing part and I thought I kind of had hoped that would be a little bit longer ‘cause that to me was the most useful part of that training.

(P5, Female, US, FI)

Indeed, some updates in the current interview guidelines, such as the ABE, seem necessary, especially on the advised techniques to develop rapport and age-adapted recommendations:

We used to have ABEs, which I don’t know if you know about. Some forces had it. Which is, you know, we haven’t used it for about five years. But that was quite limiting and didn’t really build rapport and again, prior to that would just sort of relied on intermediaries and [and] hope that we would get a bit of a chat with the child when the intermediary was doing their assessment […] I would say the oldest that I’ve used on [*ABE*] is 14 year old, but I’ve only used bits of it and again, they weren’t that happy with.

(P3, Female, UK, PO)

#### 3.2.2. Sub-Theme Two: Enhancing Understanding of Internal and External Influences

As professional child interviewers, understanding how to develop efficient rapport with the interviewee is crucial for a smoother interview process and building a trustful relationship:

*The whole thing about rapport just needs to change the way it was taught to me […] this whole thing of, you know, right before the interview starts, I’m just gonna ask you what your favourite football team is and what you had for dinner last night. You know you’ve got someone who’s sat across from you, who’s nervous and anxious and worried about having this conversation with you because, you know, there might be outwards tell you something they’ve never told anyone else before, or certainly not in the detail. They’re gonna, you know, they’ve sat there thinking what I’m here to talk about, how my granddad sexually abused me […] So, I think that just needs to change and the emphasis needs to come on the [the] rapport needs that [that] is, you know, they’re from the first moment you meet that child.*

(P2, Male, UK, PO)

Similarly, minors’ neurodevelopmental conditions (e.g., autism spectrum disorder [ASD]) could be better addressed in training to enhance understanding and ensure adaptive questioning:

*I would say a lot of our officers would [would] look at it, a child they don’t know with autism, and think that they were misbehaving or being naughty or not listening to be quite honest. I don’t think we get any training on how to deal with autistic children or children with [with] extra needs [….] I think extra training is needed because I think if you’re not used to that and you’re trying to ask questions to a child and they’re climbing on the couch behind you and they’re jumping off and they’re walking around and they’re platting your hair or [or] touching you and you’re not used to that, I think it probably would take you by surprise a bit.*

(P11, Female, UK, PO)

Another area for improvement is developing a broader understanding of children’s use of technology and how it may shape their perceptions of online crime and experiences of cybervictimisation:

*…for today’s generation where we do have social media and we do have over sexualised exposure to things and kids are more sexually active […] I’m curious to kind of see how that plays out and our kids understanding the same way we think they are. With this new generation, I think also maybe more how we can screen things like exposure to pornography, how we can ask kids about that and we can [INT: Umm] like this extortion stuff is crazy and we kind of do all that we can to gather that kind of information. But I think, I don’t know, I would like a better understanding of how to better interview kids of digital crimes. I think sometimes it can just be, it can be tricky because a lot of the victims don’t see themselves as a victim because there was nothing hands on.*

(P8, Female, US, FI)

Further comments were made on the current sexual behaviours of some young people involving the use of technology, emphasising the need for updated knowledge to enable smoother and more informed interactions:

*We did have an older lady here until she retired, and she was lovely, but I think sometimes people felt embarrassed about telling an older lady what had happened to them. Especially things like revenge porn, which is a bit of a more recent offence although it’s been around for a while now. And I think younger people understand the sort of OnlyFans*^4^ *are here, if you like, and selling things, wherever they think that older generation struggle with that.*

(P11, Female, UK, PO)

Some participants highlighted the need for professionals to make greater use of evidence when dealing with minors involved in digital crime investigations:

*I do see, like, when Homeland Security comes and does forensic interviews with us, they typically do present a lot of evidence ‘cause they are taking more of those like Internet crimes against children cases and [and] so [INT: yeah] I’m glad to see them do it. But I think like the local law enforcement, we can, we could utilise that a lot [a lot] more.*

(P7, Female, US, FI)

#### 3.2.3. Sub-Theme Three: Improving Interview Room Ambiance and Features

Participants expressed the importance of upgrading interview room features for child interviews. This would likely promote a warmer and more inviting environment for children who are asked to discuss difficult topics:

Because the other one in *LOCATION*, you know, is this old police house that just looked like any normal house but it was really cold and heating was never on […] You’d ask people to wait in the front room of this house and it just didn’t feel very welcoming. There were some toys there but not loads, and they’re all kind of outdated.

(P2, Male, UK, PO)

The necessity to develop specific adaptive room features that would help children feel more comfortable while being interviewed was commonly emphasised:

*I think the paint colour can really set the mood for the room. I think they should have something comfortable to sit in. I think both chairs should be eye level to each other so that, you know, we’re on the same eye level. In our room in particular, we have very minimal furniture. We have a few soundproofing designs on the wall, but they’re not like an actual picture if that makes sense. And then we have a very colourful rug and a table in between the two chairs, and that’s all we have in there.*

(P4, Female, US, FI)

It seems that the characteristics of the interview room may even affect courtroom proceedings and put into questions children’s statements, overall affecting their right for justice:

*…I think if you have a place that’s very distracting or has like pictures of animals or an ocean, I’ve found that kids will bring that into their disclosure. So, when I was doing interviews somewhere else, we had, like, a big ocean scene on the wall and the younger kids would be like [INT: Ohh] well, it’s like being at the beach and when the waves are moving you. And they would bring that stuff into the disclosure. And when it came to defending that in court, defence was always like, well, clearly they’re not living in reality. They’re talking about fantasy. And so how can we believe them? So, I think that can be problematic. So, it’s just like a fine line of comfortable and not sterile while not being distracting as well.*

(P8, Female, US, FI)

Participants commonly discussed strategies to improve the quality of investigative interviews with children. Key recommendations included expanding and strengthening interviewer training, particularly through longer practical sessions involving observation, role-play and feedback to enhance interviewer confidence and technique. Participants also highlighted the need to update existing professional guidance, such as the ABE guidelines (Ministry of Justice, 2022), to better address rapport-building and age-appropriate communication. Both the NICHD Protocol (Lyon, 2014) and ABE guidelines (Ministry of Justice, 2022) consider the impact of victimisation on children’s potential reluctance to discuss abuse, such as ‘The lifestyle of and the trauma experienced by some victims of and witnesses to complex cases such as child sexual exploitation and abuse is such that extensive rapport building might be needed before they develop enough trust and confidence in the investigation team to participate in an investigative interview’. Moreover, the 10-Step Investigative Interview (Lyon, 2021) recommends that interview transition to disclosure (step 7) does not directly ask questions about the abuse but rather begins with statements such as ‘Tell me why you came to talk to me’. Phase 2 of the ABE guidelines (Ministry of Justice, 2022) also recommends ‘free-narrative account of incident(s)’; however, it is also clearly stated that an explanation of the reason for an interview should be given in the first phase of the interview.

Nonetheless, some differences exist between the guidelines, such as ABE recommending four phases of the interview structure while Lyon (2021) recommends 10 steps. Both condensed rapport to a ‘phase’ or ‘step’; in the 10-Step Investigative Interview (Lyon, 2021) rapport can be interpreted as starting from the sixth phase (‘Practice Narratives’) while ‘Phase One’ in the ABE guidelines (Ministry of Justice, 2022) is named ‘Rapport’. The ABE advises rapport to not be a lengthy process, due to witnesses potentially getting tired or confused about the purpose of the interview, which could increase their anxiety. Accordingly, Lyon (2014) advises interviewers to keep the rapport phase to around five minutes as it could be fatiguing to children (NICHD Protocol). This very much contradicts with participants’ accounts on rapport-building as a form of communication, and a continuous process employed by the interviewer and followed by the child.

Both guidelines do share similarities through establishing ‘Ground Rules’ in the ABE guidelines (Ministry of Justice, 2022) and instructions in the 10-Step Investigative Interview (i.e., ‘You’re wrong’, ‘Promise to tell me the truth’; Lyon, 2021) designed to ensure that children understand the principles of interview communication, as well as concepts such as truth and lies, often by asking them questions about themselves. The diverse needs of minors, including those with neurodevelopmental conditions, were also emphasised as an area requiring greater understanding to improve comprehension and ensure more appropriate safeguarding and questioning practices. As is included in the ABE guidelines (Ministry of Justice, 2022), ‘Children with learning disabilities tend to adapt more slowly to unusual situations than their peers. It is, therefore, likely that more time will be needed to prepare the child for the interview, and extra time might be needed for the rapport phase’. Additionally, greater awareness of digital technology and online environments was considered essential, as these increasingly shape young people’s experiences, behaviours and perceptions of victimisation. Finally, participants emphasised the importance of interview room design, noting that a comfortable yet non-distracting environment can support children’s disclosure while also protecting the credibility of their statements in court. The ABE guidelines (Ministry of Justice, 2022) specifically refer to how ‘Interview rooms should, therefore, be organised so as to minimise the opportunity for distraction’ for children with learning disabilities.

### 3.3. Theme Three: Effective Techniques for Information Gathering

Identifying effective techniques in investigative interviews is essential for eliciting accurate information and establishing the truth. It is essential to employ techniques that not only facilitate the prosecution of offenders but ensure the protection of children. Participants expressed the need to continuously adapt to children’s social and cognitive development, and their language capacities. Moreover, building rapport was highlighted as a crucial approach to both gain the trust of child victims and safeguard their well-being throughout the interview process. Using cues for clarification was praised by participants, although some were considered more effective than others.

#### 3.3.1. Sub-Theme One: Strategic Adjustments to Children’s Abilities

Participants emphasised the importance of recognising that questioning minors differs significantly from interviewing adults in terms of questioning and the ability to provide detailed information:

*You’re trying to get descriptions about where the person’s hands went on the child’s private, did it go over or under the clothes? Did it, you know? Did his fingers go inside? Were they just outside? What did his fingers do? Trying to get that information is pretty difficult sometimes, especially out of the little ones because some of them don’t really know whether the hands went inside or outside, or, you know, what exactly the hands were doing.*

(P4, Female, US, FI)

In fact, most participants expressed that interviewing younger children and adolescents requires specific adaptations to account for differences in socio-cognitive behaviours:

*…a lot of teenagers would like to be kind of treated, spoken to and treated to in the same way as an adult. So, you know, that’s the kind of how they like to be spoken to. So, you [INT: Umm] I wouldn’t want to do, you know, anything that’s too kind of infantile for them, really.*

(P2, Male, UK, PO)

This is particularly true when discussing sexual abuse details, such as genitalia, clothing placement and body position:

*But like I’ve said, if you ask in a chat a little girl [INT: Umm] how did it feel? And she said, well, it hurts my tummy. That to me, says she’s been penetrated and it’s understanding that cause she’s never gonna be able to tell me that in this one, in my vagina, she is too little. So, it’s about understanding their way of communicating to you. It’s always adapting to them. Always got to be adapting to them and that’s why it’s a really good idea to speak to parents, social care teachers, the people who see them regularly and understand their terminology, especially if they have different words that they use the, you know, children have different words.*

(P1, Female, UK, PO)

#### 3.3.2. Sub-Theme Two: Continuous Development of Rapport-Building

In some official guidelines, the process of developing rapport-building with children is often advised in the preliminary phase of interviews; however, participants explained that rapport is a continuous process:

*Again, that rapport, especially if they’re a little kid where they was, like, coached like, don’t talk to me and don’t tell this person anything, and they come in here and I’ve had [INT: Umm] and we’re like, well, I understand that you’re not supposed to tell me anything so let’s talk about something else and we’ll colour or do something different. And when we’re sitting there talking, I’ll just put little drop, little hints of questions in there and they tend to open up much more so than that.*

(P10, Female, US, FI)

Rapport-building is fundamental to establish trust between the interviewers and the interviewees, but it is also imperative to establish the child’s well-being as the foremost concern:

*Exploring their feelings instead of just not. Acknowledging them, just trying to get your, I need this, this, this and this. Like they’re a child. You can address those feelings with them. And when you show that you’re willing to follow them through those feelings, they might feel a little bit more comfortable talking to you about the harder stuff because you’ve acknowledged the feelings. That makes sense.*

(P6, Female, US, FI)

As commonly described by participants, effective rapport-building can be achieved primarily through active listening and observation:

*You know, you want to build rapport with your, the [the] child you’re talking to, but you want to mostly do it in a way where they feel safe that you’re a professional who knows what you’re doing and that you’re listening to them and listening intently. I don’t wanna have them form an attachment and then only see me once, you know. But I want them to know that I’m here for them and I’m safe. And I know how to do my job, and I’m listening to what they say.*

(P7, Female, US, FI)

Offering children choices during interviews is essential for safeguarding their well-being and reducing the risk of secondary victimisation:

*If I see that a child is starting to just give me the same answer over and over, then I realise like we’re [we’re] at a point of fatigue. And if I have more questions or a lot more information that the team wants [that the team wants] to know, that’s where we would consider pausing and, you know, taking a lunch break and coming back or coming back another day.*

(P12, Male, UK, PO)

#### 3.3.3. Sub-Theme Three: Reasonable Use of Supportive Cues

While numerous techniques can support children during interviews, participants particularly highlighted certain methods, such as drawing, as being especially effective for clarification:

*We typically will colour, we’ll sit at a little table with them and colour or draw or something like that.*

(P5, Female, US, FI)

Visual aids have been encouraged to help minors discuss details of the abuse and clarify some aspects of their testimonies:

*Body maps have used quite a lot, so it’s sort of like point to the part of the bodies that was touched.*

(P9, Male, UK, PO)

Participants further discussed the use of technology for children, including with ASD, as a consideration for future practice:

*…iPads*^5^ and stuff I had looked at one of the boards they [*officers*] had like the activity table because of COVID but I thought it was a really good idea for kids with disabilities like autism and stuff like that because it’s hard to keep them focused.

(P10, Female, US, FI)

Finally, participants recommended offering animal support (real or figurine) to comfort children during interviews:

*I have a couple of like little fidget toys in there if they feel like they need to kind of fidget with something and then I have a weighted stuffed animal. So, if they feel like they want to hold something weighted, they have that option as well […] I think the only thing we’re missing that is very helpful is the facility dogs. I’ve worked in an agency where they had two working dogs in the agency and they would join us in the forensic interviews at times if the child was feeling particularly apprehensive or resistant or reluctant, we would have one of our therapy dogs in with them and it’s, it had blown me away.*

(P4, Female, US, FI)

These different aspects summarise perceived effective techniques for gathering information during investigative interviews with children while ensuring their protection and well-being. Participants emphasised the importance of adapting questioning strategies to children’s developmental, cognitive and linguistic abilities, recognising that younger children and adolescents may communicate experiences differently and require tailored approaches. Rapport-building was also identified as a fundamental and continuous process throughout the interview, helping to establish trust, acknowledge children’s emotions and support their comfort during potentially distressing disclosures. These perceptions somewhat contrast professional guidelines that often describe rapport as a designated ‘phase’ (‘Practice Narratives’ in the 10-Step Investigative Interview; Lyon, 2021) rather than a continuous process: ‘Young children may assume that adults know everything and that adults are always right: it is essential that they are given the opportunity to correct the interviewer at assessment and during the rapport phase’ (ABE guidelines; Ministry of Justice, 2022). In the 10-Step Investigative Interview (Lyon, 2021), steps named ‘Like to do/Don’t Like to do’ and ‘Last Birthday’ are dedicated to discussing ‘neutral’ topics and prepared children for the allegation questions. Similarly, in the ABE guidelines (Ministry of Justice, 2022), ‘Rapport (engage and explain)’ is predominantly recommended to ‘briefly ask some neutral questions not related to the event which can be answered positively and, therefore, create a positive mood […] so that witnesses get used to the kind of elaborated responses that will be required later’; although the intention is to help witnesses feel more comfortable, describing this as an effort to create a positive mood in children may be inappropriate, particularly given the often highly traumatic experiences they may have endured, many of which, as reported by participants, have never been disclosed before.

Within the phase-based guidelines, some recommendations for multiple phases of rapport are provided, such as ‘Interviewers should endeavour to build a rapport with reluctant witnesses and take reasonable steps to address their concerns prior to the interview. In some instances, it might be necessary to build rapport over several sessions’ (ABE guidelines; Ministry of Justice, 2022). However, some contradictory advice highlights rapport as a continuous, rather than a phase-based, process: ‘In any event, rapport should not be regarded as something that is confined to the first phase of an interview; it begins when the interviewer first meets the witness and continues throughout the interview’ (ABE guidelines; Ministry of Justice, 2022). Another aspect to point out is how rapport is recommended: ‘Some witnesses may be unhappy or feel shame or resentment about being questioned, especially on personal matters. In the rapport phase, and throughout the interview, the interviewer should convey to the witness that they have respect and sympathy for how the witness feels’; in this excerpt, rapport is not conveyed as an authentic and genuine interest in the witnesses’ stories but rather a technical tool to gather information. This does not align with participants’ emphasis on being truly interested in the child’s accounts and adapting to them to offer an easier interview process. Finally, the ABE guidelines (Ministry of Justice, 2022) recommend a closure phase, for which ‘The interviewer should always try to ensure that the interview ends appropriately. Every interview must have a closing phase. In this phase it may be useful to discuss again some of the ‘neutral’ topics mentioned in the rapport phase.’ Thus, although rapport is considered as a ‘step’ or ‘phase’ it is here suggested that it can also be used in the final phase of the interview, highlighting the lack of clarity surrounding rapport-building in the current guidelines.

Additionally, participants discussed the use of supportive cues and tools, such as drawing, body maps and technology, to help children clarify details and express themselves more effectively. As specified by the ABE guidelines (Ministry of Justice, 2022), ‘Drawings, pictures, photographs, symbols, dolls, figures and props should be used with caution and never combined with leading questions. An essential safeguard is to let the child assign meaning to any props used, to check that this meaning is stable over time, and to work with their own words or labels’. Comfort aids, including fidget toys or therapy animals, were also viewed as valuable strategies for reducing anxiety and facilitating communication during the interview process. Some caution regarding their use is explained in the ABE guidelines (2022; Ministry of Justice, 2022): ‘Children’s interactions with such dolls alone are unlikely to produce evidence that could be used in criminal proceedings’.

## 4. Discussion

The current study used hybrid thematic analysis to explore the effective and ineffective techniques and areas for improvement in current child investigative interviewing. The hybrid approach was adopted based on the need to lower subjectivity in a study aimed at adapting child interview guidelines. Through qualitative interviewing, police officers and forensic interviewers disclosed what works, what does not and how to improve child investigative interviewing. The current study’s findings can be interpreted through a conceptual understanding linking interview techniques to psychological processes and, subsequently, to interview outcomes. For instance, techniques such as rapport-building, open-ended questions and supportive interviews were reported by participants as fundamental for eliciting accurate and comprehensive disclosures from children. These techniques operate by influencing psychological processes, including memory retrieval, reduced suggestibility, emotional comfort and attention regulation (Almerigogna et al., 2007; Bull & Milne, 2020; Kim et al., 2020; Lamb et al., 2008; Lyon, 2014; Hershkowitz et al., 2017; Saywitz et al., 2015, 2019). The study demonstrates how practical interview strategies are theoretically grounded and directly connected to measurable forensic outcomes, connecting conceptual understanding with practical application. The collective evidence is discussed subsequently.

Adapting to children’s cognitive and social abilities has been proven essential for effective communication between children and adults. Previous research has found age-related increases in children’s narrative abilities and verbal performance (Bruck & Melnyk, 2004; Ornstein et al., 2004), which should be considered by professionals when interviewing young victims, especially during recall of emotional and/or traumatic events. Children are often aware of the repercussions that such allegations may have on their lives and on family members (Alaggia, 2010; K. London et al., 2005; Malloy et al., 2007). Although children may be willing to share information about abuse, they may show reluctance due to the inner turmoil between sharing the truth and fear of repercussions. Offenders may influence the child’s understanding of abusive situations by normalising the abuse, or children may be in denial and justify the abusive behaviours due to the offender’s grooming process (Winters & Jeglic, 2022). Furthermore, victims may thus feel that their behaviours do not match with common assumptions about sexual abuse, a concept highlighted in rape myths (Lonsway & Fitzgerald, 1994; Williams, 1984). For example, a common survival instinct is when victims ‘freeze’ during sexual assault, a concept described as ‘tonic immobility’ (Mann, 2023), which contradicts held expectations of how victims respond to abuse, such as showing physical resistance. These pervasive assumptions may impact victims’ willingness and abilities to report abuse, due to the fear of not being believed or not fitting the expectation of how a victim ‘should’ react (Stewart et al., 2024).

Therefore, participants perceived that rapport-building supports children’s comfort and trust, which may facilitate free recall and reduce suggestibility. This aligns with previous research demonstrating an increase in truthful accounts by victims when efficient rapport was established (Kim et al., 2020). As supported by Lamb et al. (2008), Lyon (2014), and Hershkowitz et al. (2017), adapted-related questions (such as open-ended prompts) promote free recall memory, help avoid transitioning quickly between topics and favour discussing events in chronological order. Moreover, they allow for more detailed accounts and stronger rapport with child victims, as prioritising open-ended questions can encourage children’s freedom of speech whilst also avoiding influencing victims in their accounts.

As described by participants, an approach to mitigate the use of leading questions is alternative communication techniques (drawing, visual cues) which can help facilitate more accurate and unbiased information exchange. Whilst not eliminating the risk of increased false reports of touching, studies specific to child investigative interviews specify that body diagrams can lead to higher amounts of information being gathered (Poole et al., 2011). Human figure drawings were shown to increase the number of relevant details, especially for young children (ages 4–7) and towards the end of the interview (Aldridge et al., 2004). Drawing has been correlated with richness in children’s testimonies on people, actions and locations, and it has been recommended to allow children the option to draw while recounting their experiences (Katz et al., 2018). Recent research concluded that drawings led to a higher number of details reported by children in free recall and sometimes in cued recall, as long as no suggestive interviewing prompts were used by interviewers to avoid errors and suggestibility (Derksen & Connolly, 2023). Indeed, drawings allow children to create their own retrieval cues (Allen & Butler, 2020), which could increase interviewers’ success in collecting important information during an investigation. However, it may be suggested to avoid interpreting drawings to minimise the risk of confirmation bias (e.g., instead focusing on open-ended questions can help children elaborate on what they drew).

A participant suggested that, given its successful application during the COVID-19 pandemic, the use of technology could be a valuable approach for future practice. Empirical support comes from Lim et al. (2024) who found that video interviews enabled building rapport with children, whereas the presence of parents interfered with the interview quality. Doherty-Sneddon and McAuley (2000) summarised by explaining that the positive effects of video interviews are due to social distance between the interviewer and the child, while the negative effects are linked to a reduction in visual cues. This latter finding, however, may not reflect modern technological advancements in webcam and screen quality. In childcare settings, the use of technology has been found to enhance children’s self-esteem, confidence and communication skills (Canavan Corr, 2006). Another aspect explored by researchers is the use of robots for child investigative interviews (Henkel & Bethel, 2017). Bethel et al. (2016) found that children experiencing bullying were more willing to share specific information, such as being teased about their looks, with the robot than the human interviewer. Consequently, technology-assisted interviews with children may promote cooperation and empower them to clarify or correct information when needed, an avenue that should be explored further.

During the interviews, it was proposed to use stuffed animals and real/figurine dogs for comfort and reassurance. Research has previously identified the benefits of animal-assisted therapy in children with ASD, who demonstrated increased social interaction, decreased social isolation, increased language capabilities and decreased problem behaviours compared to a group that was treated in the absence of an animal (O’Haire, 2013). Contrasting this, Côté et al. (2024) found that children who were accompanied by a dog during an investigative interview showed more signs of reluctance, even after accounting for child age and interviewer support. Therefore, facility dogs may be beneficial particularly for children who struggle verbalising their feelings and thoughts, those with developmental problems (Zilcha-Mano et al., 2011) and those with ASD (M. D. London et al., 2020). Future research should establish more conclusive findings in relation to the use of animals in child investigative interviewing.

Understanding effective interview techniques is crucial, but participants also perceived the importance of recognising challenges that may hinder their successful application. Although closed-ended questions can be used to gather specific details, relying on these questions may be leading, calling into question the integrity of the information gathered. As Davies et al. (2008) explain, children are often aware of the high status of interviewers and may be reluctant to contradict them. Therefore, reducing the use of closed-ended questions may assure that all information provided is from the children, minimising the interviewers influence and increasing the credibility of the child’s statements in court proceedings.

Despite the constraints that interviewers may face, they should provide space for children to tell their stories. Asking several questions in a row may be tempting but due to the emotions that abuse encompasses, providing space may be the solution for reflective listening (Shiau et al., 2024). Summarising what the child said by using their own words rather than paraphrasing may help mitigate potential misunderstandings between interviewers and interviewees, and aid children’s recognition of concerns and values (Braillon & Taiebi, 2020). This aligns with participants’ comments on providing time and space for victims as most of them may have experienced several types of abuse (poly-victimisation; Morlat & Alison, 2024), which may require multiple interviews to uncover.

In addition to understanding psychological mechanisms, it is important to account for the potential psychological influence of third-party presence in the interview room as this may affect the dynamics of the interview. In some countries, including the UK, an ‘Appropriate Adult’^6^ or an ‘Interview Supporter’ may be present during the interview, such as a parent/legal guardian. Participants expressed that this can be a significant distraction to children and/or interviewer and can deeply affect the interview process. Cultural expectations may influence expectations and opinions on their child being interviewed (Hritz et al., 2015). Social Judgment Theory (Sherif & Hovland, 1961) indicates that people appraise situations with their preferred position, with attitudes varying depending on their judgements of the situation. Thus, if parents are present in the interview room, they may be more likely to intervene in what the child is saying since culture pressure is stronger during adulthood than childhood. Furthermore, Interdependence Theory (Rusbult & Van Lange, 2003) suggests that because family members’ outcomes are interconnected, one member’s distress can contribute to increased stress or anxiety among other members. Therefore, a parent showing signs of dissatisfaction towards what their child is saying to the interviewer may induce feelings of pressure to accommodate the parent and potentially give false information. According to Hritz et al. (2015), false report is either a conscious process in which a child will inaccurately report claims due to pressures or an unconscious assimilation of incorrect suggestions made by people who have access to the child. Thus, due to the considerable influence of parents, not allowing their presence in the interview room could minimise the potential contamination of children’s answers and thus reduce misinformation that could possibly affect the investigation. This is an aspect that should be further explored as it contradicts official professional interviewing guidelines (i.e., ABE; Ministry of Justice, 2022).

Participants also suggested several strategies that could enhance the effectiveness of child investigative interviewing, ensuring that the process becomes as reliable as possible. Results include many statements related to the need for extended and more specialised child interview training, especially on rapport-building. As supported by research, repetitive training sessions demonstrated consistent impact on investigative interview behaviours among police officers (Akca et al., 2021). Testing and practice (‘testing effect’) have been shown to benefit long-term retention (Karpicke & Roediger, 2008). Thus, more research should measure pre- and post-training effectiveness (including role-play, feedback, observations) for child investigative interviewers.

Based on participants’ perceptions, interviewers should be made aware of the influences of the current hyper digital environment in which children are immersed (Presta et al., 2024). According to the Office of Communications (Office of Communications, 2024), 96% of people aged 3–17 in the UK went online in 2023, highlighting the centrality of the Internet in children’s lives. Similarly, in the US, nearly all teenagers (96%) say that they use the Internet everyday (Pew Research Center, 2025). A recent study from Ofcom (Office of Communications, 2025) found that 8% of minors aged 8 to 14 in the UK had accessed online pornography in the space of one month, including around 3% of 8–9-year-olds. Thus, new restrictions as part of the Online Safety Act, designed to protect children, have changed the law in the UK by requiring pornography websites to implement strict age verification checks from July 2025 (Office of Communications, 2025). As a participant mentioned, it is important to note that exposure to pornography has been identified as a risk factor in the emergence of harmful sexual behaviour for minors. Ybarra et al. (2011) claim that for minors ‘intentional exposure to violent x-rated material over time predicted an almost 6-fold increase in the odds of self-reported sexually aggressive behavior’ (p. 1). Children may not fully comprehend the effects of digital exposure on themselves and may engage in risky online/in-person behaviour, thus becoming vulnerable to online perpetration (National Society for the Prevention of Cruelty to Children [NSPCC], 2024). Indeed, recent research concluded that despite a wish to report abuse, feelings of guilt and shame were barriers to disclosure for child victims of technology-assisted child sexual abuse (TA-CSA); nonetheless, knowledge about TA-CSA was found to facilitate disclosure from the victims (Joleby et al., 2024). This highlights the potential need for further training on TA abuse and its consequences on minors, including during investigative interviews.

**Figure 3:**
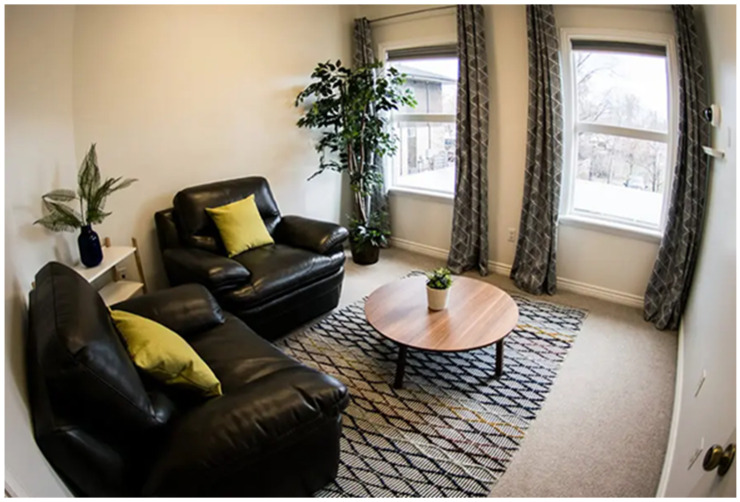
Example of a child-adapted forensic interview room. *Note*: From *Forensic Services*, by Utah County Children’s Justice Center, 2025 (https://cjc.utahcounty.gov/forensic (accessed on 6 October 2025)). CC BY-NC-ND 2.0.

Lastly, most participants commented on the importance of the interview room in developing rapport and minimising distractions. Being surrounded by a child-adapted and relaxing environment can help victims to feel at ease and be more open to discussion. Since interviewers may appear intimidating, potentially discouraging correction and disagreement (American Professional Society on the Abuse of Children [APSAC] Taskforce, 2012), an environment that promotes comfort may contribute towards assuring the well-being of children (see Figure 3).

### Strengths, Limitations and Future Directions

The current study allowed for an updated understanding of professionals’ opinions on current child investigative interviews. Since the numbers of crimes against children have increased (National Society for the Prevention of Cruelty to Children [NSPCC], 2023, 2024), developing evidence-based practice appears crucial. The current study helped to identify the techniques that work for effective interview practice and those that should be avoided. Furthermore, this study highlighted areas for improvement that could guide future research to ensure that police officers and forensic interviewers are provided with the most efficient skills for interviewing child victims, subsequently increasing successful prosecution of offenders.

The inherent limitations of the study should be noted. This study should be considered an exploratory qualitative investigation, aimed at understanding professional perceptions of child investigative interviewing rather than producing generalisable conclusions. The use of a qualitative method may constrain the strength and transferability of the findings, particularly without data triangulation. Therefore, the findings should be interpreted with caution. Furthermore, the absence of inter-rater reliability may limit the robustness of the coding. Participants were professionally based in the UK or the US, and therefore the data gathered may not be generalisable to non-Western and/or non-Anglophone countries. Owing to the small sample size, this study is exploratory and intends to serve as a complementary contribution rather than a definitive account. Additionally, data were based on self-reported experiences, which may be subject to recall bias, social desirability effects or selective reporting, limiting the internal validity and transferability of the findings. While TA highlighted common themes across participants, it may have underrepresented idiosyncratic perspectives, which could limit the depth of understanding of unique experiences. Another aspect to consider is that child abuse may be conceptualised differently in other countries and that therefore the expectations of investigative interviewing may not equate to those in the UK and the US. Thus, the results should be interpreted cautiously and are best framed as insights to guide future research involving larger samples and observations.

Future research should focus on gathering testimonies from minors and adults who have been interviewed when under the age of 18 as part of a criminal investigation. This could further develop the current findings by incorporating interviewees’ perspectives. Moreover, updated quantitative-based analysis could assist in identifying the techniques that lead to efficient interview practice on a large scale. This would help to pinpoint moments of efficient and inefficient interactions between interviewers and child victims.

Based on the themes identified in this study, several hypotheses are proposed:

- *Rapport hypothesis*: Continuous rapport-building throughout the interview will lead to more detailed narrative recall compared to rapport limited to the initial phase.
- *Age-specific guidelines hypothesis*: Adoption of age-specific guidelines will increase rapport-building and lead to higher disclosure from children.
- *Neurodevelopmental hypothesis*: Informed adaptive techniques to children’s neurodevelopmental needs will improve the completeness of their disclosures.
- *Supportive cues hypothesis*: Use of drawing or visual aids for clarification will increase the completeness of children’s disclosures.
- *Animal support (real or figurine)*: Providing animal support to child interviewees will contribute to higher disclosure.
- *Third-party hypothesis*: Absence of parents or legal guardians in the interview room will reduce false or constrained disclosures.
- *Digital awareness hypothesis*: Interviewers trained in technology-assisted abuse will elicit increased gathering of information relevant to cases involving online victimisation.
- *Interview room hypothesis*: Child-adapted interview rooms will increase children’s engagement during the interview process.

Testing these hypotheses quantitatively would allow researchers to validate the effectiveness of interview techniques, update guidelines and ultimately improve child protection through a more efficient pursuit of justice.

## 5. Conclusions

The current study shed light on a topic of great importance to protect children and hold abusers accountable: what works, what does not and how current child investigative interviewing could be improved. Through qualitative interviewing, police officers and forensic interviewers noted several limitations in the interview process, such as limited flexibility in addressing minors’ needs, the effects of abuse on children and the influence of a third party’s presence during interviews. Participants further suggested improvements including extended and more specialised training on rapport-building and technology-assisted abuse. There also appears to be a need for a deeper understanding of how technology is utilised in interviews and the impacts of digital technology on the behaviour of minors. Furthermore, participants commented on the importance of improving interview room features. They further discussed factors essential for efficient interviews, including strategic adjustments to children’s abilities, continuous rapport-building and use of supportive cues for clarification. The analysis aimed to explore patterns in practitioners’ perceptions rather than produce generalisable conclusions. This research aims to contribute to the improvement of child investigative interviews, which will further the overarching goals of increased protection of children and holding offenders accountable through more efficient prosecution.

## Figures and Tables

**Table 1:** Approach of developing TA according to Braun and Clarke’s (2006) six-phase guide.

Step	Approach
Familiarisation with the data	Interviews were read without any form of coding to develop an overall understanding of the data. Familiarising with the materials was believed to be necessary to establish an ‘overview’ of the dataset.
Generating initial codes	Systematically coding salient features in the dataset while organising data corresponding to each code.
Searching for themes	An assemblage of data into potential themes, with groupings based on their similarities in meanings.
Reviewing themes	Scrutinisation of how all the ‘groups’ of data extracted from coding were relevant to the research question. A ‘thematic map’ was drawn to help discard themes from the analysis.
Defining and naming themes	Similarities and familiarities were found between the initial themes, and three main themes were developed: *challenges and limitations in interview process, strategies for enhancing interview quality, and effective techniques for information gathering.*
Producing the report	Once the final themes were produced, articles related to the topic were used in relation to the qualitative analysis. The themes were reviewed a final time and the researchers started writing up.

## Data Availability

The datasets presented in this article are not readily available because ethical approval was granted based on the data being confidential. Access to the interview guide is restricted to researchers with appropriate training to ensure ethical and responsible use. Requests to access the interview guide and datasets should be directed to p.morlat@liverpool.ac.uk.

## Notes

1 Excerpt from the *Interview Supporters* Section in the ABE in Criminal Proceedings: ‘2.221 Right 2 of the statutory Code of Practice for Victims of Crime (Ministry of Justice, 2020) makes it clear that victims have the right to bring a person of their choice to the interview unless that is not possible, in which case victims should be told why. The guidance for service providers that accompanies the Code says that interview plans should take account of the need for an interview supporter.’

2 Research participants’ names were changed to numbers (P4 is Participant 4).

3 Interjections.

4 Launched in 2016, OnlyFans is a subscription-based platform that enables creators to share content and is most widely recognised for hosting adult material, including sexually explicit images and videos.

5 iPads are touchscreen tablet computers designed and manufactured by Apple Inc.

6 Additional information on ‘Appropriate Adult’ is accessible at https://www.gov.uk/government/publications/guidance-for-appropriate-adults/being-an-appropriate-adult (accessed on 12 October 2025).

## Abbreviations

The following abbreviations are used in this manuscript:

ABE	Achieving Best Evidence
FI	Forensic Interviewer
LD	Learning Disability
NICHD	National Institute of Child Health and Human Development
NSPCC	National Society for the Prevention of Cruelty to Children
PO	Police Officer
RP	Revised Protocol
SP	Standard Protocol
TA	Thematic Analysis
TA-CSA	Technology-Assisted Child Sexual Abuse
UK	United Kingdom
US	United States

## Appendix A. Excerpts from Lead Author’s Reflexive Diary^7^

*After interviewing participant x* *Today I realised that participants discussed the concept of rapport differently while invoking the same thing. Some refer to it as ‘positive’ interaction, while others discuss the specific technique used, often at the start of the interview. While they might all share a common understanding, I have felt that some participants think of rapport as something to ‘tick’ rather than a form of communication. Moving on, I will be aware of that when asking questions about rapport building and the overall ability to develop trust with children.*		*Thoughts on etymology:* *While searching definitions for the word ‘rapport’, I have found that the word is originated from the French verb ‘rapporter’. Being my mother tongue, this made even more sense on how to interpret it. In French, we use it to mean ‘to bring back’ (an object for example) or ‘to refer to’ (such as a discussion). ‘En rapport’ signifies ‘related to/in relation to [a topic/a person]’. We even use ‘rapport’ to define ‘a report/evaluation/summary of information’ often with the aim of sharing with others. By developing rapport, we are indirectly developing an interaction that will carry a message, a thought, an opinion. And in the context of investigative interviewing, in the hope of gathering insights to pass over. This is an aspect I will consider while interviewing and coding the data.*
	*positive interaction* *effective communication* *pre-substantive phase of interview*	*apportare (Latin) -> apporter/rapporter/être en rapport (French) -> rapport (English)*

## References

[B1-behavsci-16-00592] Abbe A., Brandon S. E. (2014). Building and maintaining rapport in investigative interviews. Police Practice & Research: An International Journal.

[B2-behavsci-16-00592] Akca D., Larivière C. D., Eastwood J. (2021). Assessing the efficacy of investigative interviewing training courses: A systematic review. International Journal of Police Science & Management.

[B3-behavsci-16-00592] Alaggia R. (2010). An ecological analysis of child sexual abuse disclosure: Considerations for child and adolescent mental health. Journal of the Canadian Academy of Child and Adolescent Psychiatry.

[B4-behavsci-16-00592] Aldridge J., Lamb M. E., Sternberg K. J., Orbach Y., Esplin P. W., Bowler L. (2004). Using a human figure drawing to elicit information from alleged victims of child sexual abuse. Journal of Consulting and Clinical Psychology.

[B5-behavsci-16-00592] Alison L., Alison E. (2017). Revenge versus rapport: Interrogation, terrorism, and torture. The American Psychologist.

[B6-behavsci-16-00592] Alison L., Alison E., Noone G., Elntib S., Waring S., Christiansen P. (2014). The efficacy of rapport-based techniques for minimizing counter-interrogation tactics amongst a field sample of terrorists. Psychology, Public Policy, and Law.

[B7-behavsci-16-00592] Allen M. L., Butler H. (2020). Can drawings facilitate symbolic understanding of figurative language in children?. The British Journal of Developmental Psychology.

[B8-behavsci-16-00592] Almerigogna J., Ost J., Bull R., Akehurst L. (2007). A state of high anxiety: How non-supportive interviewers can increase the suggestibility of child witnesses. Applied Cognitive Psychology.

[B9-behavsci-16-00592] American Professional Society on the Abuse of Children [APSAC] Taskforce (2012). Forensic interviewing in cases of suspected child abuse.

[B10-behavsci-16-00592] Banister P., Willig C., Stainton-Rogers W. (2017). Forensic psychology. The SAGE handbook of qualitative research in psychology.

[B11-behavsci-16-00592] Bethel C. L., Henkel Z., Stives K., May D. C., Eakin D. K., Pilkinton M., Jones A., Stubbs-Richardson M. (2016). Using robots to interview children about bullying: Lessons learned from an exploratory study. Proceedings of the 2016 25th IEEE international symposium on robot and human interactive communication (RO-MAN), New York, NY, USA, November 17.

[B12-behavsci-16-00592] Boyatzis R. E. (1998). Transforming qualitative information: Thematic analysis and code development.

[B13-behavsci-16-00592] Braillon A., Taiebi F. (2020). Practicing “reflective listening” is a mandatory prerequisite for empathy. Patient Education and Counseling.

[B14-behavsci-16-00592] Braun V., Clarke V. (2006). Using thematic analysis in psychology. Qualitative Research in Psychology.

[B15-behavsci-16-00592] Brown D. A., Lamb M. E., Lewis C., Pipe M.-E., Orbach Y., Wolfman M. (2013). The NICHD investigative interview protocol: An analogue study. Journal of Experimental Psychology: Applied.

[B16-behavsci-16-00592] Bruck M., Melnyk L. (2004). Individual differences in children’s suggestibility: A review and synthesis. Applied Cognitive Psychology.

[B17-behavsci-16-00592] Bull R., Milne R., Pozzulo J., Pica E., Sheahan C. (2020). Recommendations for collecting event memory evidence. Memory and sexual misconduct: Psychological research for criminal justice.

[B18-behavsci-16-00592] Canavan Corr A. (2006). Children and technology: A tool for child development.

[B19-behavsci-16-00592] Cassidy H., Akehurst L., Cherryman J. (2020). Police interviewers’ perceptions of child credibility in forensic investigations. Psychiatry, Psychology, and Law: An Interdisciplinary Journal of the Australian and New Zealand Association of Psychiatry, Psychology and Law.

[B20-behavsci-16-00592] Clarke V., Braun V. (2017). Thematic analysis. The Journal of Positive Psychology.

[B21-behavsci-16-00592] Côté É, Cyr M., Brillon P., Dion J., Daignault I. V., Gendron A. (2024). Facility dogs during police investigative interviews: Does it decrease children’s reluctance?. Child Abuse & Neglect.

[B22-behavsci-16-00592] Creswell J. W. (2012). Qualitative inquiry and research design: Choosing among five approaches.

[B23-behavsci-16-00592] Davies G., Hollin C., Bull R. (2008). Forensic psychology.

[B24-behavsci-16-00592] Derksen D. G., Connolly D. A. (2023). Drawing conclusions: Instructing witnesses to draw what happened to them. Journal of Investigative Psychology and Offender Profiling.

[B25-behavsci-16-00592] Doherty-Sneddon G., McAuley S. (2000). Influence of video-mediation on adult-child interviews: Implications for the use of the live link with child witnesses. Applied Cognitive Psychology.

[B26-behavsci-16-00592] Etikan I., Musa S. A., Alkassim R. S. (2016). Comparison of convenience sampling and purposive sampling. American Journal of Theoretical and Applied Statistics.

[B27-behavsci-16-00592] Fereday J., Muir-Cochrane E. (2006). Demonstrating rigor using thematic analysis: A hybrid approach of inductive and deductive coding and theme development. International Journal of Qualitative Methods.

[B28-behavsci-16-00592] Finlay L. (2002). Negotiating the swamp: The opportunity and challenge of reflexivity in research practice. Qualitative Research: QR.

[B29-behavsci-16-00592] Gardner H., Randall D. (2012). The effects of the presence or absence of parents on interviews with children. Nurse Researcher.

[B30-behavsci-16-00592] Harverson J., Paatsch L., Anglim J., Horwood S. (2025). Digital technology use and well-being in young children: A systematic review and meta-analysis. Computers in Human Behavior.

[B31-behavsci-16-00592] Henderson H. M., Lamb M. E. (2019). Does implementation of reforms authorized in Section 28 of the youth justice and criminal evidence act affect the complexity of the questions asked of young alleged victims in court?. Applied Cognitive Psychology.

[B32-behavsci-16-00592] Henkel Z., Bethel C. L. (2017). A robot forensic interviewer: The BAD, the GOOD, and the undiscovered. Proceedings of the companion of the 2017 ACM/IEEE international conference on human-robot interaction.

[B33-behavsci-16-00592] Hershkowitz I., Ahern E. C., Lamb M. E., Blasbalg U., Karni-Visel Y., Breitman M. (2017). Changes in interviewers’ use of supportive techniques during the revised protocol training. Applied Cognitive Psychology.

[B34-behavsci-16-00592] Hershkowitz I., Lamb M. E. (2020). Allegation Rates and credibility assessment in forensic interviews of alleged child abuse victims: Comparing the revised and standard NICHD protocols. Psychology, Public Policy, and Law.

[B35-behavsci-16-00592] Hershkowitz I., Orbach Y., Lamb M. E., Sternberg K. J., Horowitz D. (2006). Dynamics of forensic interviews with suspected abuse victims who do not disclose abuse. Child Abuse & Neglect.

[B36-behavsci-16-00592] Hritz A. C., Royer C. E., Helm R. K., Burd K. A., Ojeda K., Ceci S. J. (2015). Children’s suggestibility research: Things to know before interviewing a child. Anuario de Psicología Jurídica [Yearbook of Legal Psychology].

[B37-behavsci-16-00592] Humann M., Alison E., Alison L., Surmon-Böhr F., Ratcliff J., Christiansen P., Tejeiro R. (2023). Motivational interviewing in child sexual abuse investigations: Approaches shown to increase suspect engagement and information gathering during police interviews. International Journal of Police Science & Management.

[B38-behavsci-16-00592] Israel M., Hay I., Gadd D., Karstedt S., Messner S. F. (2012). Research ethics in criminology. Research ethics in criminology.

[B39-behavsci-16-00592] Johansson S., Stefansen K., Bakketeig E., Kaldal A. (2018). Collaborating against sexual abuse: Exploring the nordic barnahus model.

[B40-behavsci-16-00592] Johnson D. R., Scheitle C. P., Ecklund E. H. (2021). Beyond the in-person interview? How interview quality varies across in-person, telephone, and skype interviews. Social Science Computer Review.

[B41-behavsci-16-00592] Joleby M., Landström S., Lunde C., Jonsson L. S. (2024). “But I wanted to talk about it”: Technology-assisted child sexual abuse victims’ reasoning for delayed disclosure. Child Protection and Practice.

[B42-behavsci-16-00592] Karpicke J. D., Roediger H. L. I. (2008). Critical importance of retrieval for learning. Science (American Association for the Advancement of Science).

[B43-behavsci-16-00592] Katz C., Klages A.-L., Hamama L. (2018). Forensic interviews with children: Exploring the richness of children’s drawing and the richness of their testimony. Children and Youth Services Review.

[B44-behavsci-16-00592] Kim S., Alison L., Christiansen P. (2020). Observing rapport-based interpersonal techniques to gather information from victims. Psychology, Public Policy, and Law.

[B45-behavsci-16-00592] Klein M., Dorsch C., Hemmens C. (2020). Talk to me: An analysis of statutes regulating police interviews of child victims. Juvenile & Family Court Journal.

[B46-behavsci-16-00592] Koch T. (1994). Establishing rigour in qualitative research: The decision trail. Journal of Advanced Nursing.

[B47-behavsci-16-00592] Korkman J., Otgaar H., Geven L. M., Bull R., Cyr M., Hershkowitz I., Mäkelä J.-M., Mattison M., Milne R., Santtila P., van Koppen P., Memon A., Danby M., Filipovic L., Garcia F. J., Gewehr E., Gomes Bell O., Järvilehto L., Kask K., Volbert R. (2024). White paper on forensic child interviewing: Research-based recommendations by the European Association of Psychology and Law. Psychology, Crime & Law.

[B48-behavsci-16-00592] Lamb M. E., Hershkowitz I., Orbach Y., Esplin P. W. (2008). Tell me what happened: Structured investigative interviews of child victims and witnesses.

[B49-behavsci-16-00592] Lim C. A. M., Lim C. A. M., Kaveri G. (2024). Listening to children’s voices: Reflections on methods, practices and ethics in researching with children using zoom video interviews. Qualitative Research Journal.

[B50-behavsci-16-00592] Lissak G. (2018). Adverse physiological and psychological effects of screen time on children and adolescents: Literature review and case study. Environmental Research.

[B51-behavsci-16-00592] London K., Bruck M., Ceci S. J., Shuman D. W. (2005). Disclosure of child sexual abuse: What does the research tell us about the ways that children tell?. Psychology, Public Policy, and Law.

[B52-behavsci-16-00592] London M. D., Mackenzie L., Lovarini M., Dickson C., Alvarez-Campos A. (2020). Animal assisted therapy for children and adolescents with autism spectrum disorder: Parent perspectives. Journal of Autism and Developmental Disorders.

[B53-behavsci-16-00592] Lonsway K. A., Fitzgerald L. F. (1994). Rape myths. Psychology of Women Quarterly.

[B54-behavsci-16-00592] Lyon T. D. (2014). Interviewing children. Annual Review of Law & Social Science.

[B55-behavsci-16-00592] Lyon T. D. (2021). Ten step investigative interview (version 3).

[B56-behavsci-16-00592] Malloy L. C., Lyon T. D., Quas J. A. (2007). Filial dependency and recantation of child sexual abuse allegations. Journal of the American Academy of Child and Adolescent Psychiatry.

[B57-behavsci-16-00592] Mann B. (2023). Rape and social death. Feminist Theory.

[B58-behavsci-16-00592] Mertens D. M. (2010). Research and evaluation in education and psychology: Integrating diversity with quantitative, qualitative, and mixed methods.

[B59-behavsci-16-00592] Mertens D. M. (2020). Research and evaluation in education and psychology.

[B60-behavsci-16-00592] Mertens D. M. (2021). Transformative research methods to increase social impact for vulnerable groups and cultural minorities. International Journal of Qualitative Methods.

[B61-behavsci-16-00592] Ministry of Justice (2020). Code of practice for victims of crime in england and wales.

[B62-behavsci-16-00592] Ministry of Justice (2022). Achieving best evidence in criminal proceedings: Guidance on interviewing victims and witnesses, and guidance on using special measures.

[B63-behavsci-16-00592] Miragoli S., Camisasca E., Di Blasio P. (2017). Narrative fragmentation in child sexual abuse: The role of age and post-traumatic stress disorder. Child Abuse & Neglect.

[B64-behavsci-16-00592] Misirli A., Fotakopoulou O., Dardanou M., Komis V. (2025). The impact of touchscreen digital exposure on children’s social development and communication: A systematic review. Frontiers in Psychology.

[B65-behavsci-16-00592] Morlat P. V., Alison L. (2024). Understanding “Childhood Poly-Victimization” to help uncover abuse during child investigative interviewing: A systematic review. Frontiers in Psychology.

[B66-behavsci-16-00592] National Society for the Prevention of Cruelty to Children [NSPCC] (2023). 106% increase in child cruelty and neglect of-fences in England in the Past 5 Years.

[B67-behavsci-16-00592] National Society for the Prevention of Cruelty to Children [NSPCC] (2024). Online grooming crimes against children increase by 89% in six years.

[B68-behavsci-16-00592] Office of Communications (2024). Children and parents: Media use and attitudes report 2024.

[B69-behavsci-16-00592] Office of Communications (2025). Age checks for online safety—What you need to know as a user.

[B70-behavsci-16-00592] O’Haire M. E. (2013). Animal-assisted intervention for autism spectrum disorder: A systematic literature review. Journal of Autism and Developmental Disorders.

[B71-behavsci-16-00592] Ornstein P. A., Haden C. A., Hedrick A. M. (2004). Learning to remember: Social-communicative exchanges and the development of children’s memory skills. Developmental Review.

[B72-behavsci-16-00592] Pew Research Center (2025). Teens and internet, device access fact sheet.

[B73-behavsci-16-00592] Piaget J., Inhelder B. (1967). The child’s conception of space.

[B74-behavsci-16-00592] Poole D. A., Bruck M., Pipe M. E. (2011). Forensic interviewing aids: Do props help children answer questions about touching?. Current Directions in Psychological Science.

[B75-behavsci-16-00592] Presta V., Guarnieri A., Laurenti F., Mazzei S., Arcari M. L., Mirandola P., Vitale M., Chia M. Y. H., Condello G., Gobbi G. (2024). The impact of digital devices on children’s health: A systematic literature review. Journal of Functional Morphology and Kinesiology.

[B76-behavsci-16-00592] Price E. A., Ahern E. C., Lamb M. E. (2016). Rapport-building in investigative interviews of alleged child sexual abuse victims. Applied Cognitive Psychology.

[B77-behavsci-16-00592] Price H. L., Roberts K. P. (2007). A practical guide to interviewing child witnesses. The Canadian Journal of Police and Security Services.

[B78-behavsci-16-00592] Rusbult C. E., Van Lange P. A. M. (2003). Interdependence, interaction and relationships. Annual Review of Psychology.

[B79-behavsci-16-00592] Saywitz K. J., Larson R. P., Hobbs S. D., Wells C. R. (2015). Developing rapport with children in forensic interviews: Systematic review of experimental research. Behavioral Sciences & the Law.

[B80-behavsci-16-00592] Saywitz K. J., Wells C. R., Larson R. P., Hobbs S. D. (2019). Effects of interviewer support on children’s memory and suggestibility: Systematic review and meta-analyses of experimental research. Trauma, Violence & Abuse.

[B81-behavsci-16-00592] Sherif M., Hovland C. I. (1961). Social judgment: Assimilation and contrast effects in communication and attitude change.

[B82-behavsci-16-00592] Shiau A. Y. A., McWilliams K., Williams S. (2024). The role of wait time during the questioning of children: A systematic review. Trauma, Violence & Abuse.

[B83-behavsci-16-00592] Short M. A., Blunden S., Rigney G., Matricciani L., Coussens S., Reynolds C. M., Galland B. (2018). Cognition and objectively measured sleep duration in children: A systematic review and meta-analysis. Sleep Health.

[B84-behavsci-16-00592] Stewart S., Willmott D., Murphy A., Phillips C. (2024). “I thought I’m better off just trying to put this behind Me”—A contemporary approach to understanding why women decide not to report sexual violence. The Journal of Forensic Psychiatry & Psychology.

[B85-behavsci-16-00592] Surmon-Böhr F., Alison L., Christiansen P., Alison E. (2020). The right to silence and the permission to talk: Motivational interviewing and high-value detainees. The American Psychologist.

[B86-behavsci-16-00592] UK Government (2022). Challenging victim blaming language and behaviours when dealing with the online experiences of children and young people.

[B87-behavsci-16-00592] Utah County Children’s Justice Center (2025). Forensic services *[Photograph]*.

[B88-behavsci-16-00592] VanMeter F., Henderson H., Konovalov H., Karni-Visel Y., Blasbalg U. (2023). Children’s narrative coherence in “achieving best evidence” forensic interviews and courtroom testimony. Psychology, Crime & Law.

[B89-behavsci-16-00592] Wiederhold B. K. (2020). Children’s screen time during the COVID-19 pandemic: Boundaries and etiquette. Cyberpsychology, Behavior and Social Networking.

[B90-behavsci-16-00592] Williams L. S. (1984). The classic rape: When do victims report?. Social Problems.

[B91-behavsci-16-00592] Willig C., Stainton-Rogers W. (2017). The SAGE handbook of qualitative research in psychology.

[B92-behavsci-16-00592] Wilson D. B., Lipsey M. W. (2001). The role of method in treatment effectiveness research: Evidence from meta-analysis. Psychological Methods.

[B93-behavsci-16-00592] Winters G. M., Jeglic E. L. (2022). Sexual grooming: Integrating research, practice, prevention, and policy.

[B94-behavsci-16-00592] Ybarra M. L., Mitchell K. J., Hamburger M., Diener-West M., Leaf P. J. (2011). X-rated material and perpetration of sexually aggressive behavior among children and adolescents: Is there a link?. Aggressive Behavior.

[B95-behavsci-16-00592] Zilcha-Mano S., Mikulincer M., Shaver P. R. (2011). Pet in the therapy room: An attachment perspective on animal-assisted therapy. Attachment & Human Development.

